# A neuronal death model: overexpression of neuronal intermediate filament protein peripherin in PC12 cells

**DOI:** 10.1186/1423-0127-19-8

**Published:** 2012-01-17

**Authors:** Wen-Ching Lee, Yun-Yu Chen, Daphne Kan, Chung-Liang Chien

**Affiliations:** 1 Department of Anatomy and Cell Biology, College of Medicine, National Taiwan University, Jen-Ai Road, Taipei, 100, Taiwan; 2 Center of Genomic Medicine, National Taiwan University, Jen-Ai Road, Taipei, 100, Taiwan

## Abstract

**Background:**

Abnormal accumulation of neuronal intermediate filament (IF) is a pathological indicator of some neurodegenerative disorders. However, the underlying neuropathological mechanisms of neuronal IF accumulation remain unclear. A stable clone established from PC12 cells overexpressing a GFP-Peripherin fusion protein (pEGFP-Peripherin) was constructed for determining the pathway involved in neurodegeneration by biochemical, cell biology, and electronic microscopy approaches. In addition, pharmacological approaches to preventing neuronal death were also examined.

**Results:**

Results of this study showed that TUNEL positive reaction could be detected in pEGFP-Peripherin cells. Swollen mitochondria and endoplasmic reticulum (ER) were seen by electron microscopy in pEGFP-Peripherin cells on day 8 of nerve growth factor (NGF) treatment. Peripherin overexpression not only led to the formation of neuronal IF aggregate but also causes aberrant neuronal IF phosphorylation and mislocation. Western blots showed that calpain, caspase-12, caspase-9, and caspase-3 activity was upregulated. Furthermore, treatment with calpain inhibitor significantly inhibited cell death.

**Conclusions:**

These results suggested that the cytoplasmic neuronal IF aggregate caused by peripherin overexpression may induce aberrant neuronal IF phosphorylation and mislocation subsequently trapped and indirectly damaged mitochondria and ER. We suggested that the activation of calpain, caspase-12, caspase-9, and caspase-3 were correlated to the dysfunction of the ER and mitochondria in our pEGFP-Peripherin cell model. The present study suggested that pEGFP-Peripherin cell clones could be a neuronal death model for future studies in neuronal IFs aggregate associated neurodegeneration.

## Background

Peripherin is one of neuronal intermediate filament (IF) proteins that predominantly expressed in the peripheral nervous system (PNS) and in some neuronal populations of the central nervous system (CNS) [1-3]. In developing neurons, peripherin is abundant in the growth cones and required for axonal outgrowth and maintenance [4,5]. Together with other neuronal IFs, peripherin constituted a shape-maintaining IF network in mature neurons. It was reported that peripherin and α-internexin can self-assemble or co-assemble with neurofilament protein subunits to form the filamentous structure before their translocation into the axons [6-11]. Neurofilament triplet proteins are constructed by the neurofilament light (NF-L, 61 kDa), medium (NF-M, 90 kDa) and heavy (NF-H, 115kDa) subunits, all of which shared the same central rod domain with peripherin [12,13].

Peripherin plays a crucial role in development of nervous system and it can be highly relevant to pathogenesis of neurodegenerative disorder. In amyotrophic lateral sclerosis (ALS) patients, peripherin together with neuronal IFs were detected in the majority of abnormal IF inclusion bodies in mature or aging motor neurons [14-16]. Furthermore, an up-regulation of peripherin mRNA was also found in a familial ALS case [17]. A transgenic study showed that mice overexpressing peripherin developed a late-onset motor neuron death and IF inclusions resembling axonal spheroids found in ALS patients [18]. Another study showed that cultured motor neurons microinjected with an expression vector coding for the peripherin gene evoked an apoptotic cell death [19]. Besides, a recent study indicated that overexpressing peripherin can cause defective axonal transport of type Ⅳ neurofilament proteins in cultured dorsal root ganglion (DRG) neurons from peripherin transgenic embryos [20]. These studies suggest that peripherin may be an important etiological factor in neurodegenerative diseases.

The PC12 cell line was derived from a rat pheochromocytoma of adrenal medulla [21]. In response to nerve growth factor (NGF) induction, PC12 cells differentiate into a sympathetic neuron-like phenotype. This is characterized by the display of a highly organized cytoskeleton, the synthesis of catecholamine neurotransmitters and the acquisition of neurite [22]. It has been shown that peripherin is highly expressed in the PC12 cells [2,10]. Recent studies show that PC12 cells could be applied as a good cellular model for studying the pathological role of neuronal cytoskeletons in the neuronal differentiation and cell death [22-24].

To gain a better understanding of the association between overexpression of peripherin and neuronal cell death, we examined the neurodegeneration via overexpression of peripherin in PC12 cells in this study. Biochemical, cell biology, electronic microscopy, and pharmacological approaches were applied to elucidate the neuropathological mechanisms of neuronal IF accumulation.

## Methods

### Cloning of the pEGFP-Peripherin construct

A 1.7 kb rat peripherin cDNA was cloned into the *HindⅢ *site of pEGFP-C1 vector (Clontech, Palo Alto, CA) to obtain the in-frame coding sequence with EGFP. The junction for pEGFP-Peripherin fusion protein was confirmed by DNA sequencing. The final construct was named **pEGFP-Peripherin**.

### DNA transfection and selection

For the DNA transfection, 1 ×10^7 ^cells were suspended in 0.8 ml DMEM with 35 μg of DNA prepared by a Qiagen Plasmid Kit (Qiagen, Valencia, CA). Electroporation was performed by an ECM 830 electroporator (BTX, San Diego, CA) at 225 V for a duration of 20 ms. After transfection, cells were then plated on poly-D-lysine coated dishes. Twenty-four hours after plating, G418 (300 μg/ml, Invitrogen) was added to the medium for the selection of transfected cells. After 12-day selection with G418, surviving PC12 cell colonies with green fluorescence were picked up under an inverted fluorescence microscope (Leica DM IRBE HC, Wetzlar, Germany).

### Cell culture and drug treatment

The rat pheochromocytoma PC12 cell line (ATCC CRL-1721TM) was maintained as described previously [23]. Briefly, control PC12 cells and pEGFP-Peripherin cells, a stable clone established from PC12 cells overexpressing an GFP-Peripherin fusion protein [23], were cultured in Dulbecco's modified Eagle's medium (DMEM)(Invitrogen, Carlsbad, CA) containing 7.5% fetal bovine serum (FBS), 7.5% horse serum (Invitrogen), and 1 × antibiotic/antimycotic (Invitrogen) at 37°C in a 5% CO_2 _incubator. To induce differentiation, the cells were seeded at an initial density of 7.2 × 10^5 ^cells per 60 mm dish and allowed to adhere for 2 h, then were exposed to medium containing 100 ng/ml of nerve growth factor (NGF) (R&D Systems Minneapolis, MN). The medium was replaced with fresh medium containing NGF every 2 days.

When used, the selective calpain inhibitor calpeptin (20 μM) (Calbiochem, La Jolla, CA), the caspase-9 inhibitor Ac-LEHD-CMK (20 μM) (Calbiochem), the caspase-12 inhibitor Z-ATAD-FMK (2 μM) (MBL, Nagoya, Japan) was added to the medium on day 6, with 1% DMSO being used as the vehicle control.

### Antibodies

The mouse monoclonal antibodies used were anti-α-fodrin (α-fodrin is also called α-spectrin) and anti-peripherin (Chemicon, Temecula, CA), anti-phospho-NF-H (SMI-36) and anti-phospho-NF-M (RMO55) (Upstate, Lake Placid, NY), and anti-NF-H (N52), NF-M (NN18), or NF-L (NR4) (Sigma-Aldrich). The rabbit antibodies against active form of caspase-12, caspase-9 and cleaved-caspase3 were purchased from Cell Signaling Technology (Cell Signaling, Danvers, MA). The mouse anti-β-actin monoclonal antibody used as the internal control was purchased from Novus (Novus Biologicals, Littleton, CO).

### Western blot analysis

Cells were collected and resuspended in lysis buffer (10 mM Tris, pH 7.5, 100 mM NaCl, 1 mM EDTA, 1 mM EGTA, 1% Triton X-100, 10% glycerol, 0.1% SDS, 0.5% deoxycholate with a proteinase inhibitor cocktail) for 15 minutes on ice, then the lysates were centrifuged at 14,000 g for 20 minutes at 4°C and the supernatant collected for soluble protein analysis, while the pellet was resuspended in SDS-urea buffer (6 M urea, 5% SDS) to extract insoluble proteins for NF protein analysis. The protein concentrations were measured by the Bradford protein assay (Bio-Rad, Mississaura, ON, Canada). Lysates containing 20 μg or 40 μg of protein were electrophoresed on a 8% or 15% SDS polyacrylamide gel and transferred to a polyvinylidene difluoride membrane (Millipore, Bedford, MA), which was then incubated for 1 h at room temperature with 5% skim milk in Tris-buffered saline (TBS). Proteins were detected by incubating the membranes overnight at 4°C with primary antibodies against α-fodrin, p-NF-H, NF-H, p-NF-M, NF-M (all at 1:1000); cleaved caspase-3, caspase-9, caspase-12 (all at 1:500) and β-actin (1:5000) in 5% skim milk in TBS. The blots were incubated for 1 h at room temperature with horseradish peroxidase-conjugated goat anti-mouse (AP124P) or goat anti-rabbit (AP132P) secondary antibodies (Chemicon) at a dilution of 1:2000 in TBS with 5% skim milk. Western blotting luminal reagent kits (PerkinElmer, Boston, MA) and BioMax films (Kodak, Rochester, New York) were used for detection. The contrast and brightness of scanned gel pictures from Western blot were slightly adjusted using Adobe Photoshop CS3 extended 10.0 (Adobe Systems Incorporated, San Jose, CA).

### Immunocytochemistry

The cells were fixed in 4% paraformaldehyde for 15 minutes, washed 3 times in PBS, and covered with cold 100% methanol for 10 minutes. Primary antibodies were applied overnight at 4°C, then the cells were rinsed for 5 × 3 minutes with PBS, followed by FITC-conjugated goat anti-rabbit IgG and rhodamine-conjugated goat anti-mouse IgG (Sigma-Aldrich) applied at a 1:200 dilution in PBS for 2 h at room temperature. The cell nucleus was also labeled with fluorescent Hoechst 33342 (10 μg/mL, Sigma-Aldrich). After 5 more rinses in PBS, the cells were mounted and viewed on a TCS SP5 confocal microscope (Leica).

### Assessment of cell viability

The viability of pEGFP-Peripherin cells was determined by the Trypan Blue dye exclusion assay [25]. After NGF induction for 2, 4, 6, or 8 days, cells were collected in an Eppendorf tube and washed twice in PBS, then stained at room temperature for 5 minutes with 0.2% Trypan Blue solution. The stained cells were counted on a hemocytometer under a light microscope (Leica DM IL). Cell viability was calculated as the percentage of viable cells (not labelled with Trypan Blue) in the total number of cells counted.

To evaluate the protective effects of protease inhibitors on pEGFP-Peripherin cells, the XTT assay was used. Briefly, after treatment with various inhibitors, cell viability was evaluated by the ability of mitochondrial succinate dehydrogenase in live cells to reduce XTT (sodium 3'-1-(phenylaminocarbonyl)-3,4-tetrazolium-bis(4-methoxy-6-nitro) benzene sulfonic acid) salt (Sigma-Aldrich) to XTT formazan, measured on an ELx808 Absorbance Microplate Reader (Biotek InStruments, Winooski, VT) at 490 nm with a reference correction at 630 nm.

### Measurement of the mitochondrial membrane potential (△Ψm)

The △*Ψm *was measured using the fluorescent dye, tetramethylrhodamine methyl ester (TMRE) (Invitrogen). At the end of the various treatments, the culture medium was removed and replaced by 50 nM TMRE in HEPES buffer and the sampled were incubated for 20 minutes at 37°C in a 5% CO_2 _incubator. Afterwards, the cells were washed and Triton X-100 (final concentration = 0.2%) added to lyse the cells. The fluorescence of the TMRE released from the mitochondria was measured by fluorimetric analysis using a SPECTRAmax GEMINI XS Microplate Spectrofluorometer (Molecular devices, Sunnyvale, CA) with excitation and emission wavelengths of 553 nm and 578 nm, respectively.

### Transmission electron microscopy (TEM)

Cells were fixed for 2 h at 4°C with 4% glutaraldehyde in 0.1 M cacodylate buffer, pH 7.4, then rinsed 3 times in 0.1 M cacodylate buffer, post-fixed with 1% OsO_4 _in 0.1 M cacodylate buffer, dehydrated in a graded series of ethanol (70%, 85%, 95%, and 100%), and embedded in an Epon-araldite mixture. Ultrathin sections were prepared, stained with uranyl acetate and lead citrate, and examined on a Hitachi-7100 electron microscope (Hitachi, Tokyo, Japan) equipped with an AMT cooled CCD camera (Advanced Microscopy Techniques, Danvers, MA)..

### Statistical analysis

The results are expressed as the mean ± SEM and were evaluated for significance by un-paired Student's *t*-test for matched samples. Statistical significance was established at a level of *p *< 0.05. Sigmaplot 8.0 (Systat Software Inc., Chicago, IL) was used for data processing and plotting histograms.

## Results

### Establishment of pEGFP-Peripherin stable cell lines

**Figure 1:**
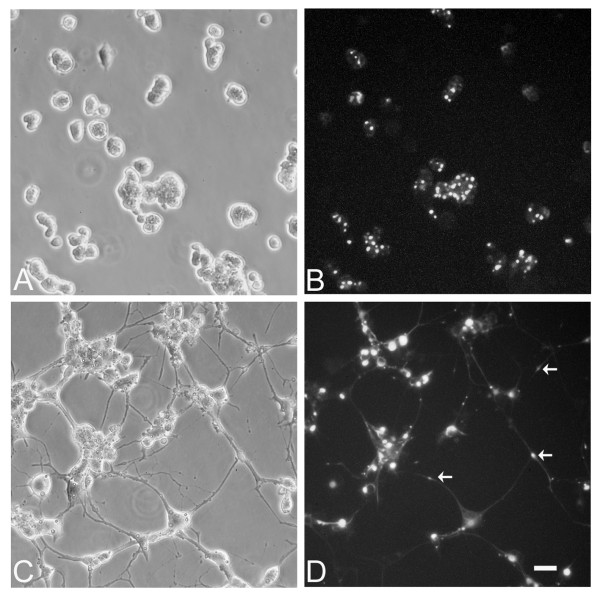
**A stable cell clone established from the PC12 cells transfected with pEGFP-Peripherin**. A pEGFP-Peripherin construct was transfected into PC12 cells by electroporation. The stable clone of transfected PC12 cells that persistently expressed the green fluorescence protein could be detected (A and B). Some accumulations of pEGFP- Peripherin proteins are found in the cytoplasm and processes of transfected cells after NGF induction for 6 days (C and D). Aggregations of EGFP-Peripherin (arrows) in the distal processes of the transfected cells could be observed (D). Scale bar = 20 μm.

To examine the effect of exogenous peripherin on neuronal IF structures and neuronal functions, the cDNA of rat peripherin tagged with enhanced green fluorescence protein (EGFP-Peripherin) was first transfected into PC12 cells by electroporation. After G418 selection, 2 stable clones were established. In our previous study [23], a stable PC12 clone expressing pEGFP was established as a control group. There were no distinguishable morphological differences between PC12 and pEGFP-transfected PC12 cells and both cells extended short neurites after NGF induction. Thus, EGFP overexpression in PC12 cells showed no effect on cell death and neural differentiation [23]. The morphology of the stable clone of pEGFP-Peripherin transfected PC12 cells under the inverted fluorescence microscope is shown in Figure 1A. Transfected EGFP-Peripehrin proteins expressed constantly and led to perikariyal aggregation in the PC12 cells (Figure 1B). After NGF induction for 6 days, transfected cells developed into neuronal phenotypes including long neurites with green fluorescence. Furthermore, protein aggregations composed of EGFP-Peripherin were also found in the cytoplasm and some cell processes (Figure 1C and 1D).

### Overexpression of peripherin induces increased expression of neuronal intermediate filaments and neurofilament hyperphosphorylation in pEGFP-Peripherin cells

**Figure 2:**
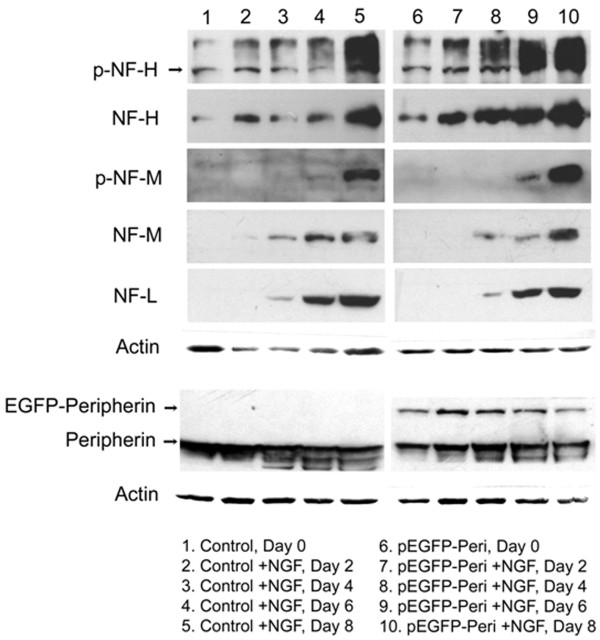
**Western blot analysis of neurofilament proteins**. Protein levels of NF-H, phosphorylated NF-H, NF-M and phosphorylated NF-M are enhanced in pEGFP-Peripherin cells, especially at 8 days of NGF induction. However, protein level of NF-L is not influenced in pEGFP-Peripherin cells. The EGFP-Peripherin fusion protein is constantly expressed in pEGFP-Peripherin cells, while not in PC12 cells. The protein level of endogenous peripherin is similar between control PC12 cells and pEGFP-Peripherin cells.

Accumulation of phosphorylated neurofilament proteins (p-NFs) in the cytoplasm or proximal axon is a hallmark of many neurodegenerative diseases, such as Alzheimer's disease and amyotrophic lateral sclerosis [26-29]. To examine whether overexpression of peripherin changed the protein level of other neuronal intermediate filaments, protein levels of nonphosphorylated and phosphorylated neurofilaments in PC12 cells and pEGFP-Peripherin cells were assayed by Western blot (Figure 2). From our observations, the protein level of endogenous peripherin was not changed between PC12 cells and pEGFP-Peripherin cells. As we presumed, the ~80 kD EGFP-Peripherin fusion protein was consistently expressed in pEGFP-Peripherin stable clones. We found that protein levels of nonphosphorylated and phosphorylated NF-H and NF-M were higher in pEGFP-Peripherin cells than that observed in PC12 cells. However, the protein level of NF-L was not significantly influenced in pEGFP-Peripherin cells (Figure 2).

**Figure 3:**
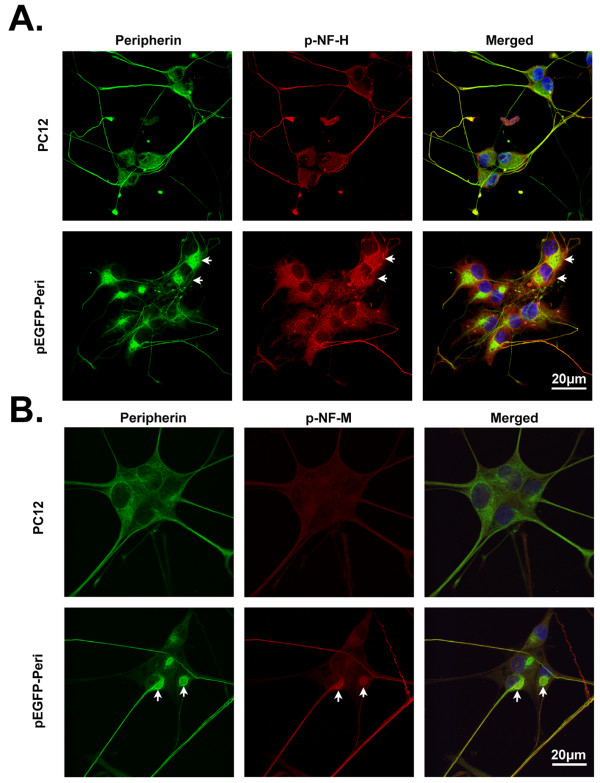
**Cellular distribution of phosphorylated NFs in PC12 and pEGFP-Peripherin cells**. (A and B) Distribution of phosphorylated NF proteins in PC12 cells and pEGFP-Peripherin cells after NGF induction for 8 days examined by immunocytochemical staining with antibodies against phosphorylated NF-H (p-NF-H) (A) or phosphorylated NF-M (p-NF-M) (B). p-NF-H and p-NF-M were detected in cytoplasmic aggregates (arrows) in pEGFP-Peripherin cells. Scale bar = 20 μm.

When the distribution of p-NFs in pEGFP-Peripherin cells was examined immunocytochemically, p-NF-H and p-NF-M were mainly observed in the neurites in control PC12 cells, whereas in pEGFP-Peripherin cells, these proteins were mainly found in the cytoplasm (Figure 3A and 3B). These data show that overexpression of peripherin not only caused increased levels of neurofilaments, but also resulted in abnormal perikaryal accumulation of phosphorylated NFs in neurons.

### Ultrastructural patterns of PC12 cells and pEGFP-Peripherin cells

**Figure 4:**
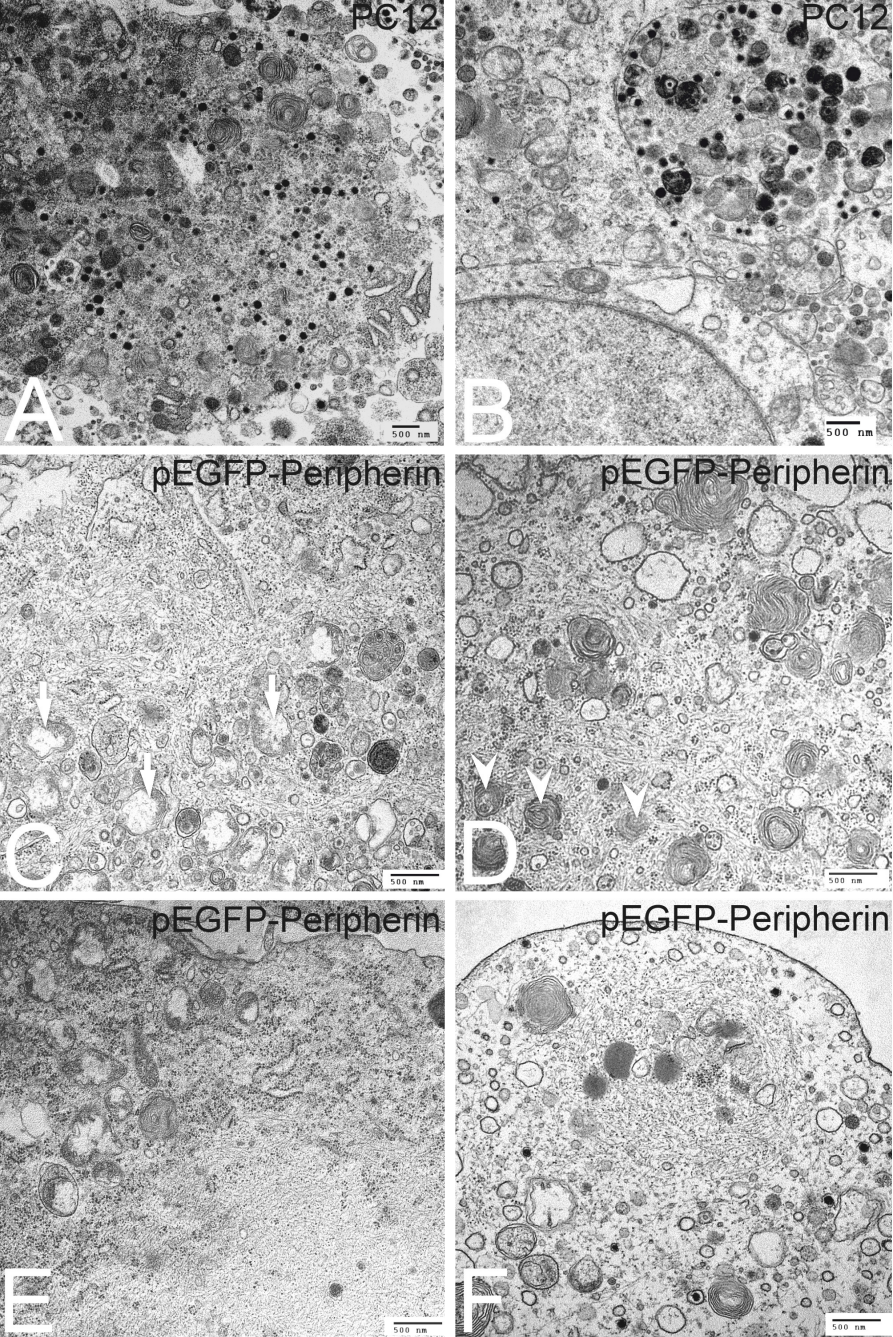
**Ultrastructural patterns of nontransfected PC12 cells and pEGFP-Peripehrin transfected cells after NGF induction for 7 days**. Ultrastructural patterns of nontransfected PC12 cells and pEGFP-Peripehrin transfected cells are examined on the 7th day of NGF induction. Many dense core granules and typical mitochondria are found in the cytoplasm (A) and processes (B) of PC12 cells. Several swelling mitochondria are observed in the cytoplasm of pEGFP-Peripherin transfected cells (C, arrow). Autophagosomes containing mitochondria are also observed in the transfected cells (D, arrowhead). Abnormal IF accumulations are found in the cytoplasm (E) and processes (F) of pEGFP-Peripherin transfected cells on the 7th day of NGF induction.

To examine if organelles were impaired in pEGFP-Peripherin cells, we investigated the ultrastructure of PC12 cells and pEGFP-Peripherin cells after NGF treatment for 8 days. Some mitochondria and dense core vesicles were found in the cytoplasm and processes of differentiated PC12 cells on the 7th day of induction (Figure 4A and 4B). Yet a few swelling mitochondria and randomly distributed IFs could be found in the differentiated pEGFP-Peripherin transfected cells (Figure 4C). Furthermore, several autophagosomes containing degraded organelles such as mitochondria were seen in some of differentiated neurons (Figure 4D). Abnormal IFs accumulations were also found in the cytoplasm of the pEGFP-Peripherin transfected cells after 7-day NGF induction (Figure 4E). Apart from cytoplasm, several electron-dense granules and a high density of IFs together with membranous organelles including mitochondria and autophagosomes were also observed in the cell process of pEGFP-Peripherin cells (Figure 4F). From these observations, we summarized that the overexpression of peripherin in the pEGFP-Peripherin transfected cells caused excrescent IFs to accumulate in the cytoplasm and neurites. Moreover, the accumulation of IF interferes with the normal functions of mitochondria and rER, and subsequently induces other death events, such as activation of the caspase cascade described below.

### Overexpression of peripherin in pEGFP-Peripherin cells induces activation of calpain, caspase-12, caspase-9, and caspase-3

**Figure 5:**
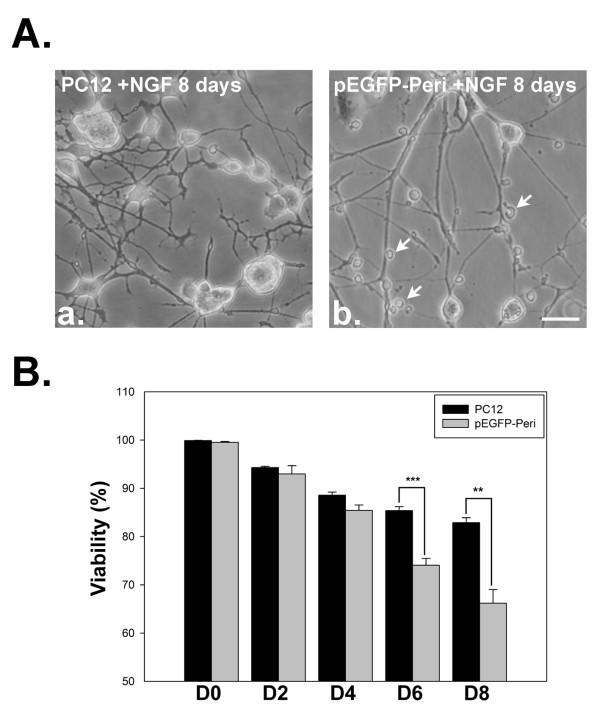
**Cell morphology and cell viability after NGF induction**. PC12 cells differentiate with neurite extension after NGF induction for 8 days (A-a). Some cell debris (arrowheads) and degenerating neurites are observed in pEGFP-Peripheirn cells after 8 days of NGF induction (A-b). (B) Cell viability assessed by Trypan blue exclusion on different days of NGF induction. * *p < 0.01, *** *p *< 0.001 in the *t*-test; n = 4. Scale bar = 20 μm.

**Figure 6:**
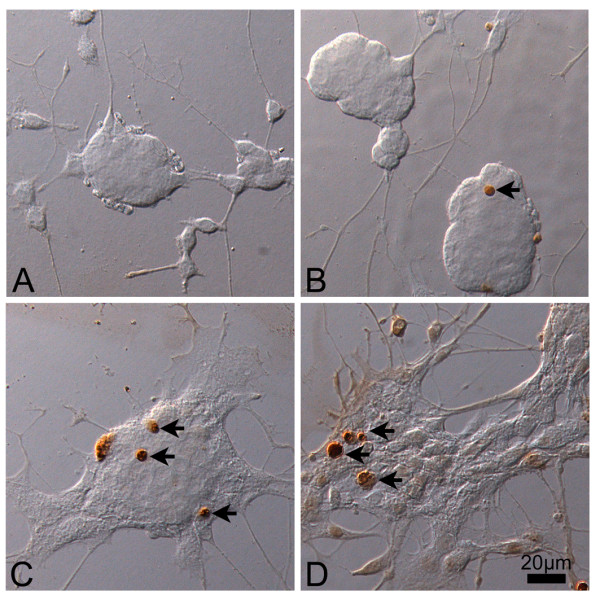
**TUNEL assays of PC12 and pEGFP-Peripherin cells after NGF induction**. TUNEL assay of control PC12 cells and pEGFP-Peripherin cells at 6 and 8 days of NGF induction. Only few TUNEL positive cells (arrows) could be found in the cell aggregation of control PC12 cells at the 6 and 8 days of NGF induction (A and B). (C) Some TUNEL positive cells could be found in pEGFP-Peripherin cells at 6 days of NGF induction. (D) More TUNEL positive cells could be detected in pEGFP-Peripherin cells after NGF induction for 8 days. Scale bar = 20 μm.

On day 8 of NGF induction in our study, well-differentiated neurons from PC12 cells were observed (Figure 5A-a), while a large amount of debris from degenerated cells was seen in pEGFP-Peripherin cells (Figure 5A-b). The viability of pEGFP-Peripherin cells decreased significantly compared to that of PC12 cells after NGF treatment for 6 days (75.06 ± 1.42% versus 85.37 ± 0.83% n = 4; *p *< 0.001) or 8 days (66.19 ± 2.81% versus 82.9 ± 1.01%, n = 4; *p *< 0.01), as shown in Figure 5B.. Due to the most neuronal cell death was through apoptosis [30,31], we used TUNEL assay to further detect apoptosis of the control and pEGFP-Peripherin cells after NGF induction. There were no detectable TUNEL positive cells found in the cell aggregation of control PC12 cells after 6 days of NGF induction (Figure 6A). Only few TUNEL positive cells were observed after 8 days NGF induction in PC12 cells (Figure 6B). Some TUNEL positive cells could be found in pEGFP-Peripherin cells at 6 days of NGF induction (Figure 6C). An increased number of TUNEL positive cells was detected in pEGFP-Peripherin cells after NGF induction for 8 days (Figure 6D).

**Figure 7:**
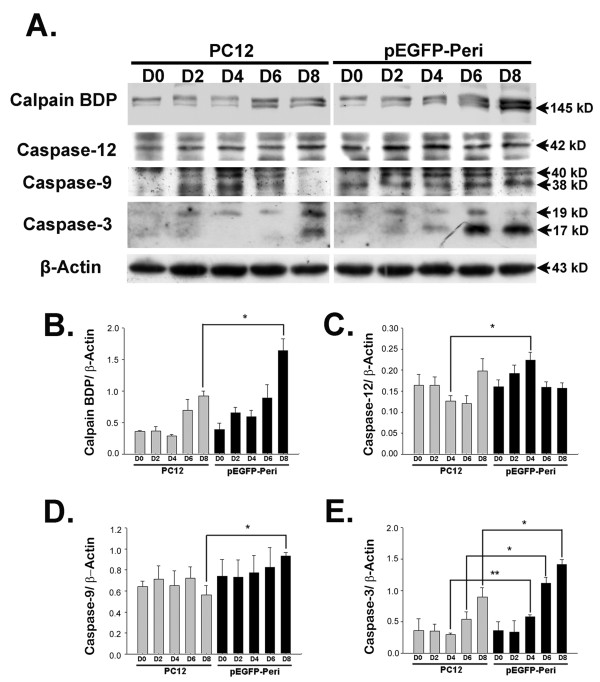
**The activity of calpain, caspase-12, caspase-9, and caspase-3 is increased on 8 day of NGF induction in pEGFP-Peripherin cells compared to PC cells**. (A) Western blot analysis showing increased activation of caspase-12 (42 kD), caspase-9 (40 kD and 38 kD), and caspase-3 (19 kD and 17 kD) on later stage of NGF induction in pEGFP-Peripherin cells compared to PC cells. (B-E) Density of the bands for the calpain BDP (B), active caspase-12 (C), active caspase-9 (D), or active caspase-3 (E) relative to the β-actin band. The values are presented as the mean ± SEM for three experiments in each group. *p < 0.05, **p < 0.01 vs. control PC12 cells.

**Figure 8:**
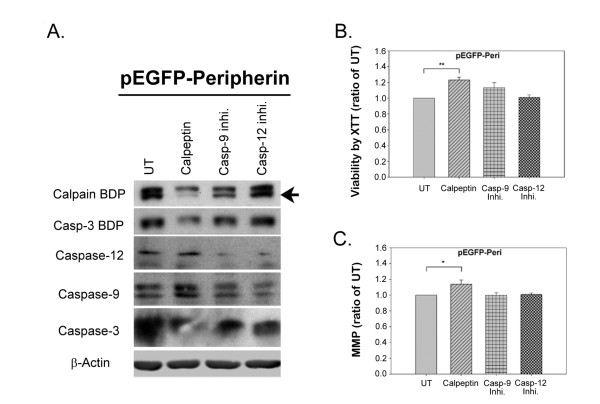
**Effects of calpain, caspase-9, and caspase-12 inhibitors on the survival of pEGFP-Peripherin cells**. pEGFP-Peripherin cells were treated for 48 h with 20 μM calpeptin (calpain inhibitor), 20 μM Ac-LEHD-CMK (caspase-9 inhibitor), or 2 μM Z-ATAD-FMK (caspase-12 inhibitor) starting on day 6 of NGF induction. (A) Proteins were analyzed by Western blotting. Caspase-3 activity was detected by levels of the 120 kD caspase-3 breakdown product of α-fodrin (caspase-3 BDP). UT = untreated group. Equal loading of protein was confirmed using β-actin. (B) Cell viability was evaluated using the XTT assay. The data are presented as the mean ± SEM for eight experiments in each group. **p < 0.01 versus untreated group. (C) The mitochondria membrane potential was evaluated by the ability of the cells to take up the fluorescent dye TMRE. Data are presented as the mean ± SEM for twelve experiments in each group. *p < 0.05 versus the untreated group.

We further investigated the activation of calpain and caspase by Western blot analysis. Functional calpain breaks down α-fodrin into a 145 kD cleavage product (calpain breakdown product; calpain BDP) and the detection of calpain BDP is performed in many studies as an indication of calpain activation [32,33]. After NGF induction for 8 days, calpain BDP levels in pEGFP-Peripherin cells were higher (1.64 ± 0.19; n = 3; *p *< 0.05) than those in PC12 cells (0.92 ± 0.08) (Figure 7A and 7B). Levels of active caspase-12 was also higher in pEGFP-Peripherin cells than in PC12 cells after 4 days (0.22 ± 0.02 versus 0.13 ± 0.01) of NGF induction (n = 3; *p *< 0.05) (Figure 7A and 7C), as were levels of active caspase-9 on day 8 (0.93 ± 0.03 compared to 0.56 ± 0.09 of control cells; n = 3; *p *< 0.05) (Figure 7A and 7D). Moreover, active caspase-3 was increased in pEGFP-Peripherin cells compared to PC12 cells on day 4 (0.58 ± 0.04 versus 0.3 ± 0.02; n = 3; *p *< 0.01) or 6 (1.12 ± 0.09 versus 0.54 ± 0.12; n = 3; *p *< 0.05) or 8 (1.42 ± 0.08 versus 0.9 ± 0.15; n = 3; *p *< 0.05) days of NGF induction (Figure 7A and 7E). The data suggests that activation of calpain, caspase 12, caspase 9 and caspase-3 is involved in the neuronal death of pEGFP-Peripherin cells. To elucidate the roles of calpain, caspase-9, and caspase-12 in pEGFP-Peripherin cells, pEGFP-Peripherin cells were treated with 20 μM Ac-LEHD-CMK (caspase-9 inhibitor), 2 μM Z-ATAD-FMK (caspase-12 inhibitor), or 20 μM calpeptin (a specific calpain inhibitor) on day 6 for 48 hours [34]. Treatment with calpeptin resulted in partial inhibition of caspase-3 activation where the production of the 120 kD fragment α-fodrin produced by caspase-3 (caspase-3 BDP) decreased (Figure 8A). Moreover, a significant inhibition of cell death was observed (123.18 ± 3.26% of levels in untreated cells; n = 8, *p *< 0.01) (Figure 8B). Calpeptin also promoted neuronal functions, such as maintaining the mitochondrial membrane potential (△*Ψm*) (113.85 ± 5.52% compared to untreated cells; n = 12, *p *< 0.05) (Figure 8C). Casepase-9 and caspase-12 inhibitors did not show significant effects on pEGFP-Peripherin cells. These results show that caspase-3 activation in pEGFP-Peripherin cells was blocked by a calpain inhibitor, which also suppressed neuronal cell death in well-differentiated pEGFP-Peripherin cells.

## Discussion

Abnormal accumulation of neuronal IFs is a conspicuous feature in many human neurodegenerative diseases [9,35-38], but the neuropathological roles of neuronal IF aggregates in the diseases are still unclear. We used the pEGFP-Peripherin cell model to study possible neuropathological pathways responsible for neurodegenerative disorders. Neuronal IF aggregates were seen in the early stages (d0-d2) of differentiation of pEGFP-Peripherin cells, while neuronal death was significantly increased in well-differentiated pEGFP-Peripherin cells (Figure 5). Interestingly, hyperphosphorylation of NFs were also observed in well-differentiated pEGFP-Peripherin cells (Figure 2 and 3). NFs, especially NF-M and NF-H, have many Lys-Ser-Pro (KSP) repeats in the C-terminal region that can be phosphorylated by Cdk5 and GSK-3β [39-48]. Phosphorylation of the C-terminal region, in particular that of NF-H, regulates NF axonal transport [49]. Extensive C-terminal NF phosphorylation-induced impairment of NF axonal transport may be due to a weak affinity for kinesin and premature NF-NF polymerization [50-52]. In our case, immunofluorescence and Western blot experiments have demonstrated that accumulation of hyperphosphorylated neurofilaments in cytoplasma of pEGFP-Peripherin cells (Figure 2 and 3). These results lead us to an assumption that overexpression of peripherin may induce the accumulation of hyperphosphorylated neurofilaments in cytoplasma that resulted from inappropriate activation of kinases. Therefore, we would like to further confirm which kinases involved in neurofilament hyperphosphorylation in pEGFP-Peripherin cells.

Transmission electron microscopy showed that not only mitochondria, but also rER and autophagosomes, were trapped in the neuronal IF aggregates in pEGFP-Peripherin cells. Interestingly, many mitochondria and regions of rER were swollen, implying dysfunction of these organelles (Figure 4C, D, E and 4F). It has been reported that the ER and mitochondria can sense stress and initiate the cell death pathway [53]. Active calpain, caspase 12, caspase 9 and caspase-3 increased and were detected in pEGFP-Peripherin cells (Figure 7). Calpain, a cysteine protease, is mainly activated by elevated intracellular calcium levels, and calpain activation is involved in necrosis and apoptosis [54,55]. Calpain can activate caspase-3, caspase-9, and caspase-12 [56-60]. Procaspase-12 can be activated by ER stress and mobilization of intracellular calcium ion stores [60]. Caspase-9 is involved in mitochondrial-induced apoptosis, and cleaved caspase-9 activates caspase-3 to initiate the caspase cascade, which leads to apoptosis [61]. In summary, active caspase-12 sequentially activates caspase-9 and caspase-3, resulting in induction of apoptosis [62]. Caspase 12 and calpain might remain activated when treating with the caspase 9 inhibitor since calpain works upstream of caspase in apoptosis [59,60] and activation of caspase 9 requires caspase 12 [63]. In our experiments, there is no significant change in the MMP and cell viability after caspase 12 or 9 inhibitor treatment. We supposed that single caspase inhibitor treatment could not prevent cell death completely. When treating with calpain inhibitor, cell death was dramatically reduced in our study. Our functional analysis confirmed that caspase-3 activation was downregulated in pEGFP-Peripherin cells by inhibitors of calpain, caspase-12, or caspase-9 (Figure 8A). We suggest that activation of calpain, caspase-12, caspase-9, and caspase-3 is implicated in neuronal death of pEGFP-Peripherin cells.

Imbalance of cellular calcium ion could be caused by ER and mitochondrial impairment and it might lead to activations of calpain and caspases in apoptosis [64,65]. In our pEGFP-Peripherin cell model, we observed abundant neuronal IF accumulation in the cytoplasm and triggers hyperphosphorylation of NF proteins, which do not transport to the axon and subsequently aggregate. Mitochondria and ER are trapped in the neuronal IF aggregates and indirectly damaged. We suggest that activation of calpain, caspase-12, caspase-9, and caspase-3 are correlated to the dysfunction of the ER and mitochondria in pEGFP-Peripherin cells.

Transgenic mice overexpressing wild-type mouse NF-H or NF-M show neither muscle atrophy nor motor neuron loss, despite prominent axonal swelling and perikaryal neurofilament accumulation in motor neurons [66,67]. However, overexpression of peripherin developed a late-onset motor neuron death and IF inclusions resembling axonal spheroids found in ALS patients [18]. Since neuronal IF accumulation is seen in the perikarya and axons of affected motor neurons in SOD1 mutant transgenic mice [68,69], this transgenic mouse model will be a good candidate for confirming the therapeutic effects of protein kinase inhibitors *in vivo*. Additionally, both the ubiquitin-proteasome system and the autophagy-lysosomal system are important in protein degradation in neuronal metabolism [70].

## Conclusions

In summary, these results suggested that the cytoplasmic neuronal IF aggregate caused by peripherin overexpression may induce aberrant neuronal IF phosphorylation and mislocation subsequently trapped and indirectly damaged mitochondria and ER. In our pEGFP-Peripherin cell model, the dysfunction of the ER and mitochondria is related to the activation of calpain, caspase-12, caspase-9, and caspase-3. The present study suggested that pEGFP-Peripherin cell clones could be a neuronal death model for future studies in neuronal IFs aggregate associated neurodegeneration. Furthermore, our pEGFP-Peripherin cell model could provide a good alternative system to the SOD1 mutant mice *in vivo *model and can be used to study the protein degradation machinery and elucidate the complex neuropathological underlying mechanisms of neuronal cell death.

## List of abbreviations

IF: intermediate filament; GFP: green fluorescent protein; NF: neurofilament; ALS: amyotrophic lateral sclerosis; DRG: dorsal root ganglion; PNS: peripheral nervous system; CNS: central nervous system; NGF: nerve growth factor; XTT: sodium 3'-1-(phenylaminocarbonyl)-3,4-tetrazolium-bis(4-methoxy-6-nitro) benzene sulfonic acid; TMRE: tetramethylrhodamine methyl ester; TEM: transmission electron microscopy.

## Competing interests

The authors declare that they have no competing interests.

## Authors' contributions

WCL and YYC carried out all experiments in the study and helped to draft the manuscript with DK. CLC conceived of the study and participated in its design and coordination as well as revised the manuscript. All authors read and approved the final manuscript.
